# Recent Developments in Nanotechnology for Detection and Control of *Aedes aegypti*-Borne Diseases

**DOI:** 10.3389/fbioe.2020.00102

**Published:** 2020-02-20

**Authors:** Estefânia Vangelie Ramos Campos, Jhones Luiz de Oliveira, Daniele Carvalho Abrantes, Carolina Barbára Rogério, Carolina Bueno, Vanessa Regina Miranda, Renata Aparecida Monteiro, Leonardo Fernandes Fraceto

**Affiliations:** ^1^Human and Natural Sciences Center, Federal University of ABC, Santo André, Brazil; ^2^São Paulo State University—UNESP, Institute of Science and Technology, Sorocaba, Brazil

**Keywords:** *Aedes aegypti*, vector control, biosensors, larvicides, arboviruses, nanobiotechnology

## Abstract

Arboviruses such as yellow fever, dengue, chikungunya and zika are transmitted mainly by the mosquito vector *Aedes aegypti*. Especially in the tropics, inefficacy of mosquito control causes arboviruses outbreaks every year, affecting the general population with debilitating effects in infected individuals. Several strategies have been tried to control the proliferation of *A. aegypti* using physical, biological, and chemical control measures. Other methods are currently under research and development, amongst which the use of nanotechnology has attracted a lot of attention of the researchers in relation to the production of more effective repellents and larvicides with less toxicity, and development of rapid sensors for the detection of virus infections. In this review, the utilization of nano-based formulations on control and diagnosis of mosquito-borne diseases were discussed. We also emphasizes the need for future research for broad commercialization of nano-based formulations in world market aiming a positive impact on public health.

## Introduction

Rampant growth of human population has led to major challenges of sustainable food production and disease control in the twenty-first century (Roni et al., 2015). Arthropods are vectors of some deadly diseases, which can lead to epidemics or pandemics (Murugan et al., 2015). On top of the list are mosquitoes (Diptera: Culicidae) that are a cause of a major concern around the world because they can act as vectors of a variety of harmful pathogens and parasites (Benelli, 2016; Benelli and Mehlhorn, 2016). *Aedes aegypti* and *Aedes albopictus* are the most important global vectors of arboviruses, such as dengue, yellow fever, chikungunya, and zika viruses (Durán et al., 2016). Arboviruses have long been treated as neglected diseases around the world. However, in recent years there have been a number of epidemics caused by arboviruses - such as dengue, chikungunya, yellow fever and unprecedented zika (Wilder-Smith et al., 2017). The main factors contributing to these outbreaks have been considered to be urbanization, modernization and increased international mobility of the general population (Tavares et al., 2018).

Control of arboviruses is difficult due to many factors, such as lack of effective vaccines for most of the arboviruses, lack of antiviral drugs, insecticide resistance in the vectors such as *Aedes* species and failure of vector control strategies that would decrease human-vector contact (Batool et al., 2018). In this scenario, development of new approaches to rapidly detect, and control dissemination of arboviruses are a priority and a public health imperative. In this regard, the importance of nanobiotechnology has been gradually realized as an emerging technology of the future due to exceptional new benefits (Suganya et al., 2017). In the vector control applications, nanoparticles could be applied for: (a) the development of new drugs, with higher activity, decreased toxicity and sustained release; (b) development of new repellent formulations based on natural or synthetic compounds; (c) control of vectors by the use of nanoparticles with repellent, insecticidal or larvicidal activities (Magro et al., 2019); and (d) development of biosensors that can rapidly detect and diagnose the mosquito transmitted viral diseases (Durán et al., 2016; Benelli et al., 2017; Nicolini et al., 2017a). Due to the lack of specific drugs for viral diseases, nanobiotechnology has appeared as an important new breakthrough, which could be potentially used for treatment of patients infected with arboviruses. VivaGel® is a poly-L-lysine dendrimer-based formulation, which has shown efficient antiviral activity against zika virus (ZIKV) (Starpharma, 2016). Recently, a number of reviews have been published on the contribution of nanotechnology to control arboviruses epidemics.

The developments in the area of nanomedicines is also promising new treatments for different diseases, improving the efficacy and bioavailability of drugs, with controlled release formulations that require optimal doses and consequently lesser adverse effects. This review discusses the current status of nanobiotechnology relevant to the control of arbovirus mosquito vectors, and highlights how it provides key tools for exploring new perspectives in the treatment of arboviruses.

## Nanotechnology for Arbovirus Detection and Control

**Figure 1:**
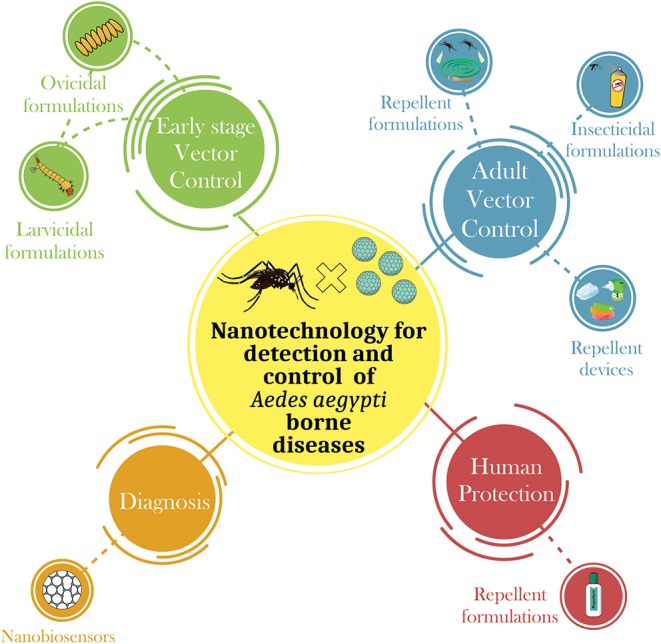
Summary of the main applications of nanotechnology in the control of *Aedes aegypti-*borne diseases. There are four main areas where nanotechnolgy can be connected: (i) *Early stage vector control*—The nanodevices for the control of *Aedes aegypti* can be developed in order to control this pest in its different stages of life. Many studies have developed nanodevices to control the early stages of mosquitoes, i.e., products that exhibit ovicidal and/or larvicidal activity; (ii) *Adult vector control—*adult insect control is also highly studied form of control through the development of nanoformulations that exhibit larvicidal and/or repellent activity; (iii) *Human protection—*following the recent epidemic of arboviruses transmitted by *Aedes aegypti*, many studies have focused on the development of forms of immunization of humans through the development of nanovaccines, and (iv) *Diagnosis—*the development of nanobiosensors that quickly detect the presence of arboviruses in the host, thus expediting the decision for the most effective treatment.

Several strategies have been applied to prevent proliferation of *Aedes* species using physical, biological, and chemical control approaches. Other methods under research and development, are also being studied, including the use of nanotechnology to produce repellent and larvicidal formulations that are more efficacious and less toxic. The development of nanotechnology-based sensors for rapid viral detection has also attracted the attention of scientific community (Figure 1).

### Biosensors

Rapid diagnosis of important arboviruses-borne diseases such as dengue, chikungunya, zika, and yellow fever is essential in order to reduce and avoid further dissemination of the infections within the general population (Patterson et al., 2016). The WHO has emphasized the importance of developing point-of-care (POC) tests that are ASSURED (Affordable, Sensitive, Specific, User-friendly, Robust and rapid, Equipment-free, and Deliverable) (Pashchenko et al., 2018). An ideal technique for the on-site detection of arboviruses should have these characteristics and enable early detection of the disease. Fast and timely diagnosis is crucial for the confirmation of viral infection so that it can be followed by clinical treatment, and necessary measures can be put in place for monitoring and protection of public health (Rashid and Yusof, 2018). Currently, diagnosis of the infections caused by arboviruses in the genus *Flavivirus* (family *Flaviviridae*) is often late, ineffective, and dependent on the clinical symptoms. The final decision on the infection generally requires a long waiting time, collection of samples from suspected patients (blood, urine, or saliva), transportation and preservation, and laboratory procedures by trained health staff.

**Figure 2:**
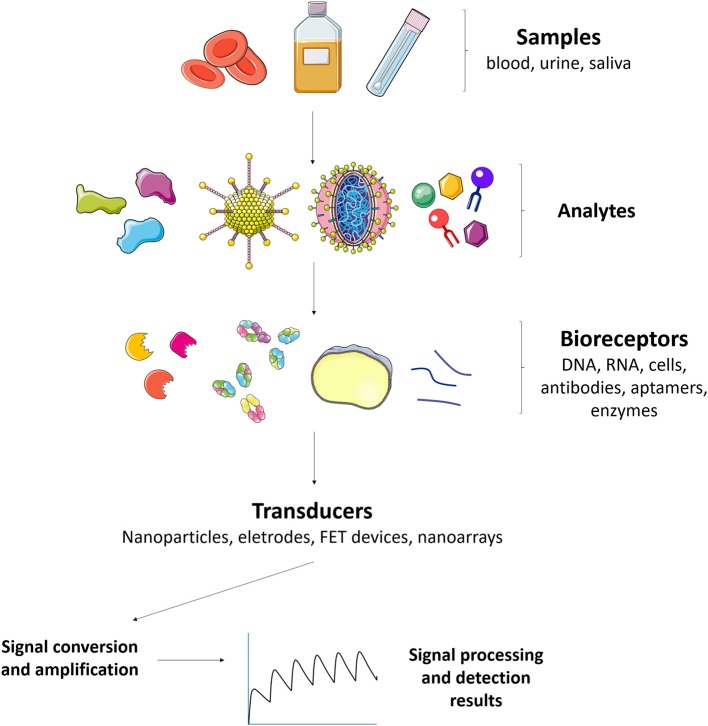
Major constituents of a biosensor for arboviruses detection and control. Different kinds of samples (blood, urine, saliva) can be used to detect analytes such as NS1 antigen and IgM antidengue, zika, and/or chikungunya antibodies. The analytes can interact with bioreceptors (aptamers, gold nanoparticles, glycan, fluorophores, enzymes, viral antigens, nucleic acids, and polyclonal antibodies for instance) that are connected to transducers and the signal converted and amplified in order to monitor the arboviruses.

Given such difficulties in the early detection of an impending epidemic of a viral infection, such as dengue, chikungunya, zika, and yellow fever (Nakata and Röst, 2015), there has been an urgent need for the improvement of existing tools and development of new biosensing technologies that are rapid, effective, and applicable in terms of real-time diagnosis. Biosensors are biologically-selective analytical devices that are able to recognize analytes in a complex sample matrix without the need for lengthy sample treatments. The biologically-selective part of biosensors enables them to produce highly specific responses by means of a transduction system that acts to convert the biological recognition into a quantifiable electrical signal (Figure 2). For the detection of arboviruses, the system must be able to identify low concentrations in complex sample matrices, such as blood, saliva, urine, and serum, without pretreatment or with minimal sample preparation.

The great advantage of a biosensor is that the bioreceptor interacts specifically with an analyte molecule. The specific interaction causes one or more physicochemical changes (production of ions, colored moieties, electrons, gases, heat, mass, or light) (Sethi, 1994). These responses can then be amplified and transformed into easily interpretable results. For the control and diagnosis of an endemic disease, an ideal biosensor device should be able to detect the arbovirus during all stages of infection, so the device must be designed to carry more than just one bio-receptor (multiplex sensing). Such features can be achieved using lateral-flow assays (LFAs) and lateral flow immunoassay (LFIA).

**Figure 3:**
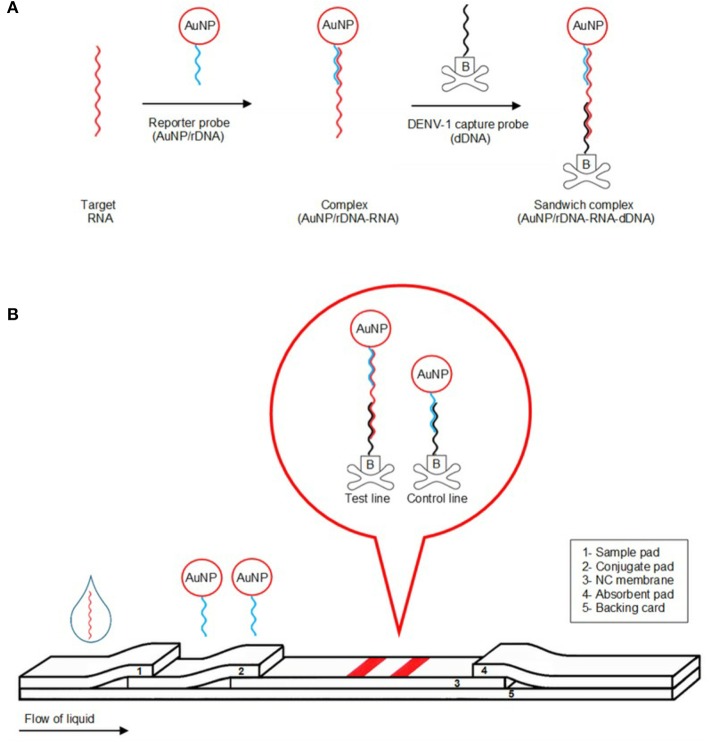
Illustration of detection mechanism of the detection of dengue-1 RNA using dextrin-capped AuNP as label in a POC device. **(A)** Formation of AuNP/rDNA-RNA-dDNA sandwich complex. **(B)** Schematic of visual detection. Reprinted with permission from Yrad et al. (2019). Visual detection of dengue-1 RNA using gold nanoparticle-based lateral flow biosensor. *Diagnostics* 9:74.

Several recent studies have used mainly LFAs as the basis for the construction of ASSURED biosensing devices. These assays can be performed using microfluidic technologies such as paper-based miniaturized devices that combine several recognition steps in a small area for naked-eye detection and quantification of compounds in complex mixtures, with the sample being collected on a test device and the results being displayed in real time (Koczula and Gallotta, 2016). Glucose, urine, and pregnancy test strips are examples of LFA devices, where the fluid containing the sample (blood, saliva, serum, etc.) moves by capillary action through various stages were antibodies and conjugated labels (nanoparticles, for instance) can interact and react to the fluid and, finally, show (sandwich assays) or not (competitive assays) a colored line at the test line position. More detailed information about LFAs can be found at (Sajid et al., 2015; Koczula and Gallotta, 2016; Carrell et al., 2019). Figure 3 provides a scheme of biosensor based on LFA where a colorimetric lateral flow biosensor (LFB) for the visual detection of dengue-1 RNA using dextrin-capped gold nanoparticle (AuNP) as label can be seen.

The detection of dengue and yellow fever has been performed with a platform for multiplexed pathogen detection employing multi-colored silver nanoplates (Yen et al., 2015) as demonstrated by Yen et al. (2015). In this study, the authors showed that the color of test lines could differentiate among different bioreceptors, with the analyses being performed in various ways, including the use of mobile phone applications. Current improvements in LFA technology are associated with the use of nanotechnological tools, such as lab-on-a-chip devices and nanoparticles that change color when aggregated. These improvements have significantly enhanced diagnosis sensitivity and selectivity.

Nawaz et al. (2018) reported a novel method for the detection, classification, and antibody screening of dengue virus, based on electrochemical impedance spectroscopy (EIS), involving protein recognition by means of a self-assembly process based on polymer matrix composites. However, as mentioned previously, it is very important to be able to achieve early detection of arbovirus infection but the biosensors had generally been designed to detect NS-type proteins that are only produced from the fifth day of infection onwards (Nawaz et al., 2018). Omar et al. (2018) overcame this limitation by designing an optical sensor based on the surface plasmon resonance phenomenon, which was applied to the diagnosis of dengue virus structural E-protein that forms the coat of the host virus itself. This protein can be detected earlier, at the start of the immune system response to the infection (Omar et al., 2018).

**Figure 4:**
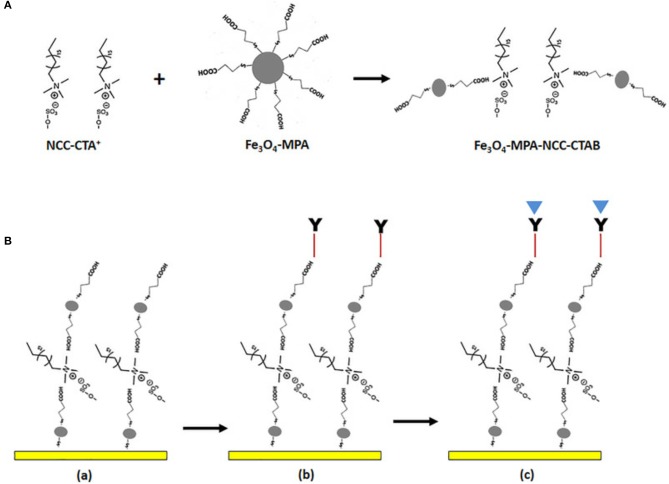
**(A)** Illustration of possible mechanism for the preparation of Fe-MPA-NCC-CTAB composite aiming the dengue virus recognition **(B)** The sensor functionalization. (a) Surface activation of gold/Fe-MPA-NCC-CTAB layer, (b) Immobilize the IgM antibody via EDC-NHS cross-linker, (d) Injection of dengue E-protein solution. Reprinted with permission from Omar et al. (2018). Development of an optical sensor based on surface plasmon resonance phenomenon for diagnosis of dengue virus E-protein, Sensing and Bio-Sensing Research 20, 16–21.

Figure 4 shows the schematic immobilization of IgM in gold/Fe-MPA-NCCCTAB (3-mercaptopropionicacid - nanocellulose crystalline/hexadecyltrimethylammonium bromide, respectively)/EDC-N-hydroxysuccinimide (NHS) for early detection of dengue virus E-protein using surface plasmon resonance explored by Omar et al. (2018). By introducing IgM immobilized Fe-MPA-NCC-CTAB/EDC-NHS on a gold surface, it is possible to determine the E-protein concentration in a range of 0.0001–10 nM. The sensitivity found by optical sensor in contact with DENV is 39.96° nM^−1^ (Omar et al., 2018).

Vinayagam et al. (2018) reported the recognition of serotype-specific DENV employing multicolor triangular silver nanoparticles (TAg), which has the potential to be a powerful diagnosis technique that is able to differentiate between various serotypes. The color responses were established on the interaction of a TAg-DNA probe with a specific strand, resulting in the creation of a network association between the DNA probe and the dengue virus RNA, according to serotype. This was the first report of DNA conjugated to triangular silver nanoparticles, based on the pH reduction method. This biosensor has not yet been tested using real samples from infected patients, although the proposed technology appears to be very promising for use in clinical POC diagnostic testing (Vinayagam et al., 2018).

Other available methodologies and R&D developments for the improvement and development of biosensing systems for the detection of arboviruses are shown in Table 1.

**Table 1:** Main biosensors developed for the detection of arboviruses, together with their operational principles and applications.

**Biosensor type**	**Disease**	LOD^*^	**Detection speed**	**Detection medium**	**References**
Electrochemical biosensor based on surface imprinted polymers	zika	2 × 10^−4^ PFU.mL^−1^	–	Buffer	Tancharoen et al., 2019
		2 × 10^−3^-5 × 10^−2^ PFU.mL^−1^		Serum	
Magneto-enzyme LFIA combining super-paramagnetic nanoparticles	DENV-1	0.25 ng.mL^−1^	90 min	Serum	Thanh et al., 2019
	DENV-2	0.1 ng.mL^−1^			
	DENV-3	0.25 ng.mL^−1^			
	DENV-4	1.0 ng.mL^−1^			
Nitrogen-doped porous carbon-based fluorescence sensor	zika RNA	0.23 nM	40–80 min	Saliva	Li et al., 2019
Paper-plastic microfluidic hybrid chip integrated with a lateral flow immunoassay	Dengue	84.66 ng.mL^−1^	<2 min	Spiked buffer	Yuzon et al., 2019
Trapezoidal SiNWs array fabricated by AFM-LAO	Dengue	2.22 fM	Real-time	DNA	Yusoh et al., 2019
Aptamer–gold nanoparticle conjugates	zika	10 ng	Real-time	*A. aegypti* salivary gland extract	Bosak et al., 2019
Acrylic-based genosensor (DNA biosensor)	Dengue (DEN-2)	1.21 × 10^−16^ M	30 min	Blood	Mazlan et al., 2019
				Urine	
				Saliva	
Gold nanoparticle-based lateral flow biosensor	Dengue	0.01 μM	20 min	Synthetic dengue-1 target	Yrad et al., 2019
		1.2 × 10^4^ PFU.mL^−1^		Pooled Human Sera	
Fluorescent lateral flow immunoassay	zika (NS1)	0.045 ng.mL^−1^	20 min	Buffer	Rong et al., 2019
		0.15 ng.mL^−1^		Serum	
Localized surface plasmon resonance immunosensors	Dengue (4 serotypes)	10^7^ TCID50.mL^−1^	<5 min	–	Basso et al., 2018
Two-dimensional MoS_2_ nanosheets-based disposable biosensor (electrochemical detection)	Chikungunya	3.4 nmol.L^−1^	<60 min	PBS serum	Singhal et al., 2018
Paper-based DNA biosensor using gold shell-coated magnetic nanocubes	Chikungunya	0.1 nmol.L^−1^	–	PBS serum	Singhal et al., 2018
Laser-cut microfluidic device made of glass-fiber paper	Non-structural 1 (NS1) viral protein and specific IgM	25 ng.mL^−1^	<10 min	Blood and plasma	Theillet et al., 2018
Graphene-based biosensor employing precise immobilized monoclonal antibody	zika	450 pmol.L^−1^	5 min	–	Afsahi et al., 2018
Electrochemical immunosensor	Dengue	0.3 ng.mL^−1^	–	Serum	Nawaz et al., 2018
Reverse-transcription LAMP coupled with reverse dot blot	zika	<2 × 10^3^ (6 RNA copies per reaction)	Between 3 and 10 min	Saliva	Sabalza et al., 2018
Multiplex tools with target-specific fluorescently tagged strand displaceable probes with RT-LAMP	Dengue	~1.22 PFU equivalent viral RNAs	30 min	Urine and plasma	Yaren et al., 2017
	zika	~0.71 PFU equivalent viral RNAs			
	Chikungunya	~38 copies of viral RNA			
Electrochemical stand with electrospun semi-conducting manganese (III) oxide (Mn_2_O_3_) nanofibers for DNA hybridization detection	Dengue	120 × 10^−21^ mol.L^−1^	–	Spiked serum	Tripathy et al., 2017
Electrochemical capacitive sensing	Dengue	0.5 ng.mL^−1^	–	Serum	Cecchetto et al., 2017
	zika				
	Chikungunya				
Coupling of reverse-transcription loop-mediated isothermal amplification (RT-LAMP) with the quenching of unincorporated amplification signal reporters (QUASR) technique	Dengue	10^3.4^ copies.μL^−1^	<40 min	Blood, urine, and saliva	Priye et al., 2017
	zika	10^5^-10^2^ PFU equivalent.mL^−1^ (10^4^-10^1^ copies per rxn) 10^8^-10^3^ PFU.mL^−1^	7–15 min <40 min		
	Chikungunya				
Surface-enhanced Raman spectroscopy (SERS)-based sandwich immunoassays (LFA)	zika (NS1)	0.72 ng.mL^−1^	20 min	Serum	Sánchez-Purrà et al., 2017
	Dengue	7.67 ng.mL^−1^			
Bead-based immunofluorescence assay on a microfluidic dielectrophoresis platform	Dengue	10^4^ PFU.mL^−1^	5 min	–	Iswardy et al., 2017
Optical caustic plasmonic light scattering sensor	Dengue	50 pg.mL^−1^	15 min	Serum	García et al., 2017
Carbon nanotube-based chemiresistor functionalized with heparin	Yellow fever	8.4 × 10^2^ TCID50.mL^−1^	10 min	–	Wasik et al., 2017
	Dengue				
Multiplexed assay on a nanostructured plasmonic gold (pGOLD) platform	zika (NS1)	0.33 IgG level	120 min	Serum	Zhang et al., 2017
		0.30 IgA level			
Reverse transcription strand invasion-based amplification (RT-SIBA) with fluorescence detection	zika	5,000 copies.mL^−1^	<30 min	Lysis buffer	Eboigbodin et al., 2016
Detection using isothermal amplification, AC susceptometry, and magnetic nanoparticles	zika virus oligonucleotide	1 aM	27 min	Serum	Tian et al., 2016
Reverse-transcription loop-mediated isothermal amplification (RT-LAMP)	zika	50–100 PFU.mL^−1^	40 min	Saliva	Song et al., 2016
Lateral flow assay using multicolored silver nanoparticles	Dengue	150 ng.mL^−1^	–	Blood	Yen et al., 2015
Carbon nanotube-ink printed electrode	Dengue	12 ng.mL^−1^	–	Serum	Dias et al., 2013
Optical DNA biosensor based on square-planar ethyl piperidine substituted nickel (II) salphen complex	Dengue	0.2 mol.L^−1^	30–120 min	Saliva and urine	Ariffin et al., 2013
Microfluidic system combined with microvalves and micropumps for rapid DNA hybridization using shuttle flow	Dengue (4 serotypes)	100 pmol.L^−1^	90 s	–	Huang et al., 2010
Microfluidic chip that accomplish DNA/RNA amplification, sample injection, and separation of nucleic acid products	Dengue	–	<5 min	–	Huang et al., 2010
Sensor-based microchip employing a magnetic bead bioassay platform	Dengue (antidengue virus IgG)	100 pg.mL^−1^	–	–	Aytur et al., 2006

* *Limit of detection*.

The recent increase in the cases of epidemic infections worldwide (Nicolini et al., 2017b) has also been matched by an increase in the available commercial biosensing devices and diagnostic methodologies. An excellent example is the invention designed by Kaushik and Nair (2018). The device is based on a modeling and electrochemical immunosensing approach for the detection of zika virus and offers an extremely low detection limit (picomolar). The biosensor is shaped to be worn on an individual's skin for infection screening. It matches the analyte measurement to the baseline in order to determine if an infection is present. After signal interpretation, the result is transmitted as an alert message. The device can be classified as a point-of-care biosensor, since it complies with the ASSURED features as proposed by the WHO.

The Ulisse Biomed SRL product portfolio includes a biosensor for the determination of an infection, and possible associated infections, caused by several viral pathogens, including zika, dengue, and chikungunya. The biosensor designed by Braga et al. provides a method for obtaining qualitative and quantitative data related to viral infections. This specific biosensor consists of an antigen (biologically active responsive element) bound covalently to the surface of one or more carbon nanotubes and/or metal nanoparticles present on part of a microelectrode surface, together with an electrically conducting part where a transduction system (sensor) converts the biochemical response into an electric signal (Braga et al., 2015).

Table 2 lists some of the published patents related to the improvement of biosensing tools and emerging new technologies for more efficient detection of analytes related to dengue, chikungunya, zika, and yellow fever.

**Table 2:** Patents related to the detection and control of arboviruses.

**Country**	**Company**	**Product technology**			
			**Year**	**Patent number**	**References**
United States	Mikrogen, GmbH	Method for the immunological diagnosis of a sample with a potential infection with an arbovirus and test kits suitable for this purpose	2019	US2019/0227065A1	Soutschek et al., 2019
United States	The USA, as represented by the Secretary, Dept. Of health and human services	Compositions and methods for the diagnosis and treatment of zika virus infection	2017	WO2018152496A1	Akahata and Ueno, 2017
United States	University of Central Florida Research Foundation, Inc. (UCFRF)	A payload reservoir comprising an insect attractant or insect food source; and (b) A detector conjugate comprising a gold nanoparticle conjugated to a specific detector molecule that binds specifically to a protein present in the saliva of a specific insect to be detected	2018	US20180231550A	Willenberg and Seal, 2018
United States	The Hong Kong polytechnic university	Microarray design of hybrid upconversion nanoparticles on a nanoporous anodized alumina membrane heterogeneous assay for simultaneous detection of multiple oligonucleotides	2018	US2018/0246084 A1	Hao et al., 2018
United States	Sympano, Inc.	Nano-field electrical Sensor for Biomarkes and other targets analytes by determining impedance in bodily fluid on nanoporous membrane		US2018/0067107 A1	Barrett et al., 2018
China/ United States	Ulisse Biomed SRL	Biosensors for the detection of infection and associated maladies	2017	CN106461667A US20170212116A1	Braga et al., 2015
United States	DexCom, Inc.	Transcutaneous analyte sensor for transcutaneous measurement of glucose in a host	2018	US7654956B2	Brister et al., 2012
Spain	Universidad complutense de Madrid	Biosensor for the detection of nucleic acids	2017	ES2580138B2	Cabarcos et al., 2017
United States	University of Ottawa, University of Malaya	Long-range surface plasmon-polariton biosensor	2018	WO2018090125A1	Berini and Wong, 2018
United States	Florida International University	Electrochemical sensing device based on nano-devices for fast zika Virus detection	2018	US10012645B2	Kaushik and Nair, 2018
United States	San Diego State University (SDSU) Foundation	Cell-based devices for track small molecules that restrain enzymes	2018	US10006077B2	Wolkowicz, 2018
United States	Eccrine Systems, Inc.	Biosensing device aimed to be used in humans skin to track an infection by one or more antigens	2018	WO2018026931A1	Beech et al., 2018
United States	Aviana molecular technologies, LLC	Multiplex acoustical biosensor with higher sensitivity	2011	US20110136262A1	Ragavan et al., 2011
United States	Purdue research foundation	Electrochemical biosensor for RNA and DNA sensing	2017	US20170107565A1	Marinero-Caceres et al., 2017
United States	Aviana molecular technologies, LLC	Biocoated piezoelectric biosensor platform for point-of-care diagnostic use	2015	US20150111765A1	Laury-Kleintop and Rutner, 2015
France/ United States	Cornell Research Foundation, Inc.	Microfluidic biosensor and methods of use	2005	WO2005084404A2	Baeumner et al., 2005
Australia/ United States	Lifeprint Australia Pty Ltd.	Auto-feedback loop biosensor—signal amplification auto-feedback loop for the detection of a target analyte in a sample	2009	WO2009152566A1	Fletcher and Milligan, 2009
United States	UT-Battelle, LLC	Biosensor which has multiple functions and broad spectrum and methods of utilization	2004	USOO6743581B1	Vo-Dinh, 2004

The advancement of technology can show how to improve the ways to detect arboviruses with nano based-tools. However, just a few publications on the use of nano-tools has been clearly demonstrated. Nanosensors have the potential to be allies in the early detection of *Aedes aegypti*-borne diseases by offering novel approaches to achieve sensitive, specific, and stable recognition in complex matrices in quick or real-time diagnostics. Although some variations in synthesis protocols can still be tested to improve the productivity and efficiency of nanomaterials for diagnostic applications, more research on the way lab-to-practical nanodiagnostics is needed. Not only more research must be done on nanosensors for *A. aegypit*-borne diseases, but the upscale of the applicability is urgently necessary, as shown by the few patents filed for this purpose (Table 2). One of the ways for this accomplishment is to narrow the scientific boundaries between disciplines such as chemistry, sociology, bio, and nanotechnology and information technology. A good example that results from this new multidisciplinary approach is the smartphones-based POC devices, as previously cited. They are a real and promising novel tool for flaviviruses detection without complex instruments, since, in a simple way, the blood sample can be analyzed under 40 min (Priye et al., 2017; Rong et al., 2019). Despite the advances, still, there is an urgent need for proper and precise use of nanosensors in hospitals, field, and to prevent these potential health risk diseases.

### Insect Repellents

The development of new nanotechnology-based formulations for the encapsulation of natural and synthetic repellents is an important strategy for obtaining systems that are more effective and have fewer undesirable impacts. These sustained-release formulations provide controlled or slow release of active agents into the environment, increasing the duration of action and reducing human exposure to the agent (for example, by permeation through the skin). Encapsulation also protects the active agent against premature degradation caused by the effects of light, temperature, oxidation, and humidity, among others (Tavares et al., 2018). Numerous matrices (synthetic and natural) can be used for the preparation of carrier systems, including polymers, proteins, lipids, polysaccharides, and others. It should be noted that the main desirable characteristics of such matrices are biocompatibility and biodegradability, as well as low cost (Barradas et al., 2016).

Gomes et al. encapsulated DEET in polymeric nanospheres, resulting in particles with an average diameter of 114 ± 37 nm, low polydispersion index, and stability as a function of time. The sustained release of nanoencapsulated DEET provided repellency for over 9 h, which was longer than obtained using free DEET. The results showed that the release mechanism was temperature dependent, which the authors highlighted as having great potential, since the release rate could be adjusted by alteration of temperature (Gomes et al., 2018).

Silva et al. (2019) encapsulated essential oils of *Piper aduncum* L. and *Piper hispidinervum* in gelatin nanoparticles and evaluated effect against *Aedes aegypti* Linn. Results showed a high encapsulation efficiency of the EOs (around 80%), average size around 100–200 nm, zeta potential around−40 mV. Both encapsulated EOs reached lethal dosages within 24 h of exposure and total mortality of the tested pests (Silva et al., 2019).

Forgearin et al. prepared and characterized permethrin-loaded lipid nanocapsules and tested their application as repellents in clothes. The formulations presented a mean particle diameter of 201 ± 4 nm, with a monomodal size distribution and permethrin content of 4.6 ± 0.1 mg/mL. It was observed that even after washing and with the action of temperature, the polyester fabrics containing the nanoparticles had higher concentrations of permethrin, compared to those containing only the free compound. The results showed that the innovative repellent spray composed of the nanoparticle formulation was useful for the impregnation of clothes and was promising for the protection of an individual against insects (Forgearini et al., 2016).

Werdin González et al. prepared and characterized polymeric nanoparticles (composed of PEG and chitosan) for the encapsulation of essential oils (geranium and bergamot). Evaluation was also made of the acute and residual larvicidal activities of the formulations against *Culex pipiens*. Physicochemical characterization showed that the PEG nanoparticles containing the essential oils had a mean size of <255 nm and provided encapsulation efficiencies between 68 and 77%, while the chitosan nanoparticles presented a mean size of <535 nm and encapsulation efficiencies between 22 and 38%. Both systems showed high larvicidal activity (acute and residual), with the chitosan-based formulations having the best effects. These findings demonstrated the potential of polymeric nanoparticles containing essential oils for use as eco-friendly larvicidal products (Werdin González et al., 2017).

Silva et al. studied the encapsulation of the essential oils of *Piper aduncum* L. and *Piper hispidinervum* C. in gelatin nanoparticles, with evaluation of the biological effects against *Aedes aegypti*. The encapsulation efficiencies exceeded 80%, and the particles were spherical, monodispersed, and smaller than 100 nm in size. Both of the encapsulated essential oils provided lethal effects within 24 h of exposure, with *Aedes aegypti* mortality greater than 80% (Silva et al., 2019).

It should be noted that the search for new formulations has also led to patenting. For example, patent **BR1020180168665** describes the preparation and characterization of nanostructured lipid carriers (NLCs) and nanoemulsions containing citronella and neem oil, for the control of insects such as *Aedes aegypti*. The results showed that both the nanoemulsions and the NLCs loaded with the essential oils caused 100% mortality of *A. aegypti* larvae during the first day of exposure, while the NLCs without essential oil induced 100% mortality after 10 days of exposure. Both carriers showed satisfactory efficacy in the control of *A. aegypti* larvae (Fraceto et al., 2018).

The patent **WO2017143421A1** describes the invention of cosmetic formulations for use as topical insect repellents, employing polymeric micelles, nanoemulsions, and solid lipid nanoparticles containing active repellent substances. Nanoencapsulation techniques were used to produce systems consisting of nanostructures with stable hydrophobic and hydrophilic chains. The formulations presented sustained release of the active substances, consequently providing long duration of action of the repellents, together with greater safety (Paula et al., 2017).

The patent **US20190160016A1** present an invention to a nanoparticle composition comprising one or more β-triketones selected from *Leptospermum scoparium* botanical extract. The prepared system presented high insecticidal activity against ecotoparasites, and according to the inventors can also be applied to repel insects like *Aedes aegypti* (Thomas, 2019)

Table 3 presents other studies concerning the use of formulations based on micro/nanotechnology for the encapsulation of compounds (natural and synthetic) presenting repellent activity.

**Table 3:** Formulations based on modified release systems for the encapsulation of active agents with insect repellent properties.

**Systems**	**Matrices**	**Active agents**	**Main characteristics**	**References**
Polymeric nanoparticles	Poly(ethylene glycol) (PEG)	Diethylphenylacetamide (DEPA)	Diameter: 149 ± 1.06 nm; Properties: 5-fold decrease of median lethal indices (LC_50_), compared to free DEPA	Balaji et al., 2017
Polymeric nanospheres	Poly(n-butyl methacrylate-co-methyl methacrylate)	N,N-diethyl-m-toluamide (DEET)	Diameter: 114 ± 37 nm. Properties: release rate of the encapsulated DEET provides repellency for over 9 h and is and more controlled when compared to the free DEET	Gomes et al., 2018
Nanoemulsion	Polaxamer 407	Ethyl butylacetylaminopropionate (IR3535)	Diameter: ± 200 nm; Properties: Nanoemulsion less retained by the epidermis and not toxic to the cells	Pinto et al., 2017
Gel/Nanoparticle	Chitosan	*Zanthoxylum acanthopodium* essential oil (ZA EO)	Encapsulation efficiency of 96.64%; Properties: Reduction in essential oil permeation in *in vitro* membrane study and mosquito repellent activity against *Aedes aegypti* with protection time of 2 h	Sharma, 2019
Polymeric nanoparticles	Polyethylene glycol	Quercetin	Diameter: 124.0 ± 1.1 nm; Properties: Stability at 4°C, affected larval *Aedes aegypti* development, less toxic than non-encapsulated quercetin toward *C. vulgaris* (green alga)	Pessoa et al., 2018
Polymeric micelles	PEG and PLGA	Pyrethrins	Diameter: 140–320 nm. Properties: Protection against ultraviolet degradation (at 26°C) and high larvicidal activity against *Culex pipiens pallens*	Zhang et al., 2018
Polymeric microparticles	Gum arabic	Essential oils and DEET	Diameter: 1–68 mm; Properties: Spherical shapes and cotton fabric impregnated with system presentend better insect repellency, compared to DEET	Eyupoglu et al., 2018
Nanoparticles	Chitosan	*Siparuna guianensis* essential oil	Diameter: 268 ± 3.4 nm; Encapsulation efficiency 84.8–88.0% Properties: 100% mortality during the first week and provides Against *Aedes aegypti* mosquito larvae	Ferreira et al., 2019
Polymeric nanoparticles	Polaxamer 407	Eugenol, 1,8-cineole, geraniol, linalool, carvacrol, α-terpineol, citronellol, thymol, and menthol	Diameter: Around 40 nm; Properties: Mortalities ranging from 30 to 60% against insects with linalool and 1,8-cineole being most effective	Lucia et al., 2017
Polymeric microparticles	Cellulose	N,N-diethyl-m-toluamide (DEET)	Encapsulation efficiency of 98%. Properties: A significant reduction in release rate of DEET	Kadam et al., 2019
Inclusion complexes	β-Cyclodextrin	*Lippia gracilis* essential oil	Inclusion complex formation by kneading and co-evaporation with essential oil content ~15%; Properties: LC_50_ of 33 ppm toward *Aedes aegypti* larvae and inclusion complex was not harmful to non-target organisms	Galvão et al., 2018
Polymeric micropartices	Carboxy-methylcellulose (CMC)	Essential oils (*Alpinia galanga, Citrus grandis*, and *C. aurantifolia*), and DEET	Diameter: 4–200 μm. Properties: The same period of repellent activity for essential oil encapsulated in comparison with microencapsulated DEET. Extended duration of repellent activity (between 1 and 2 h) compared with commercial formulations	Misni et al., 2017
Nanoemulsion	Tween 80	*Vitex negundo* L. essential oil	Diameter: <200 nm; Properties: Nanoemulsion with higher larvicidal activity (*Aedes aegypti*) compared with only essential oil;	Balasubramani et al., 2017
Nanoemulsion	Tween 80	*Ocimum sanctum* essential oil	Diameter: 50–300 nm; Properties: Nanoemulsion with potential insecticidal effect against *Aedes aegypti* and *C. quinquefaciatus* adults	Ramar et al., 2017
Nanofibrous	Cellulose	Citriodiol (CD)	Properties: Nanofibrous presented more prolonged repellency (34 days) than monolithic ones in experiments using *Aedes aegipty*	Muñoz et al., 2019

DEET is currently considered a “gold standard” due to its outstanding protection against mosquitoes and other biting insects. It is the most common active ingredient in all commercially available repellents and is used as a comparative for other substances (Khater et al., 2019). However, due to indiscriminate use has suffered resistance effects, leading to loss of formulations effectiveness. In addition, due to its toxicity has raised health and environmental concerns Thus, the search for natural alternative repellents as well as new molecules is necessary. This is the case of compound IR3535, one of the newest products with odorless and non-toxic characteristics, which is recommended for children over 6 months of age and pregnant women (Benelli et al., 2018a).

It is in this scenario of innovation and search for new solutions that nanotechnology applies. It is becoming increasingly necessary to develop formulations that increase the repellent's longevity by controlling delivery and evaporation rate. In addition, the different applications forms such as sprays, creams, lotions, aerosols, oils, adhesives, protective clothing, treated nets, among others, is very important in order to ensure options for people living in endemic areas (Tavares et al., 2018; Agnihotri et al., 2019). Thus, encapsulation in micro/nanoparticles, cyclodextrins, micelles, hydrogels among others constitutes an approach to modify the physicochemical properties of encapsulated repellents. When applied in topical formulations or in personal protective clothes, for example, they have been shown to be more effective in increasing repellency time and also in reducing dermal absorption, improving the safety profiles of these products (Ahmed et al., 2019; Osanloo et al., 2019). Innovative nanotechnology-based formulations should be followed by safety and efficacy studies, as this will increase consumer confidence in this new formulations (Hameed et al., 2019).

### Larvicidal and Ovicidal Nanoparticles

The green or biological synthesis of nanoparticles, also called biogenic synthesis, can offer advantages over the classical nanotechnological techniques currently employed. The new synthesis methods are economical, fast, and less expensive, and are performed at ambient temperature and pressure. In contrast, standard physical and chemical techniques typically involve high energy consumption, due to the need for high pressures and temperatures (Benelli et al., 2018b). In addition, potentially harmful reagents and solvent are not used in green synthesis methods, because the reducing and stabilizing agents are substituted by molecules produced by living organisms (Kumar et al., 2015). These agents can be extracted from bacteria, fungi, yeasts, algae, or plants (Sintubin et al., 2012). The technique can be used to produce nanoparticles composed of metals, metal oxides, silica, and carbon (Benelli et al., 2016).

Recent studies have reported the synthesis of various nanoparticles using different natural extracts, with demonstration of the larvicidal, ovicidal, and mosquiticidal activities of these nanoparticles. Udayabhanu et al. observed the larvicidal effect of titanium dioxide nanoparticles (TiO_2_ NPs) synthesized using an aqueous extract of *Euphorbia hirta* leaves against the larvae of *Aedes aegypti* (LC_50_ = 13.2 mg/L) and *Culex quinquefasciatus* (LC_50_ = 6.89 mg/L) (Udayabhanu et al., 2018).

Another study proposed the green synthesis of Ag NPs for use as an environmentally-friendly alternative to pyrethroid and carbamate larvicides. Silver nanoparticles synthesized from extracts of the *Quisqualis indica* plant showed high toxicity against the vectors of filariasis, zika virus, and malaria. In addition, toxicity tests employing three non-target organisms indicated low toxicity of these systems (Govindarajan et al., 2016).

The patent databases include inventions that describe methods of green synthesis of metal nanoparticles and metal oxides, with broad applications in consumer products, medicines, pharmaceuticals, and other biomedical products (Hoag et al., 2011; Liu et al., 2013; Yujia et al., 2015; Awad et al., 2016). There have been several reports concerning vector control using biogenic nanoparticles (Table 4), indicating the promising potential of these nanoparticles that are both environmentally-friendly and highly effective for vector control.

**Table 4:** Biogenic nanoparticles tested for the control of disease vectors.

**Name**	**Action**	**Organism species**	**LC_**50**_/ LC_**90**_ in** ***A. aegypti***	**Treatment time**	**References**
AgNP	Larvicidal property against fourth instar larvae of *Aedes aegypti*	Apple extract	AgNPs -T 15.76/27.7 ppm AgNPs -RT 29.81/42.3 ppm	24 h	Ali et al., 2017
AgNP	Ovicidal activity against *Aedes aegypti*	*Bauhinea acumiata* leaf powder aqueous extract	27.19/52.32 μg.mL^−1^	24 h	Alharbi et al., 2018
AgNP	Larvicidal activity against *Aedes aegypti, Anopheles stephensi*, and *Culex quinquefasciatus*	Leaf extracts of *Leucas aspera* and *Hyptis suaveolens*	4.02/11.22 mg.mL^−1^	24 h	Elumalai et al., 2017
AgNP	Ovicidal activity against *A. aegypti* eggs	*Holarrhena antidysenterica* bark extract	5.53/12.01 ppm	72 h	Kumar et al., 2018
ZnONP	Larvicidal and ovicidal activities against *Aedes aegypti*	*Scadoxus multiflorus* leaf powder aqueous extract	34.04/78.06 ppm	24 h	Al-Dhabi and Valan Arasu, 2018
ZnONP	Larvicidal activity against of *Aedes aegypti* (4th instar)	Extract of the seaweed *Ulva lactuca*	22.38/41.94 μg.mL^−1^	24 h	Ishwarya et al., 2018
ZnONP	Larvicidal activity against fourth instar of *Aedes aegypti*	*Pedalium murex* seed extract	34.88/64.56 μg.mL^−1^	24 h	Ishwarya et al., 2017
AgNP	Larvicidal property against *Anopheles stephen and A. aegypti*	*Belosynapsi Kewensis* leaf extract	84.2/117.3 ppm	24 h	Bhuvaneswari et al., 2016
AgNP	Potential larvicidal activity against larvae of *Aedes aegypti* (3rd instar), *Anopheles stephensi*, and *Culex quinquefasciatus*	Aqueous leaf extract of *Heliotropium indicum*	72.72/126.86 μg.mL^−1^	24 h	Veerakumar et al., 2014
AgNP	Larvicidal activity against third and larvae of *Aedes aegypti* (4th instar)	Leaf extract of *Derris trifoliata*	3rd instar: 7.0/17.76 mg.mL^−1^ 4th *instar*: 5.87/12.11 mg.mL^−1^	24 h	Kumar et al., 2017
AgNP	Larvicidal activity against 1st−4th instar larvae dengue vector	3,5 di-t-butyl-4 hidroxyanisole isolated from *Cynodon dactylon* leaf	1st−4th instar: 2.5; 2.78; 3.02; 3.05/8.28; 7.47;8.13;8.74 μg.mL^−1^	24 h	Ramanibai and Velayutham, 2016
AgNP	Larvicidal and pupicidal against *Aedes aegypti* and *Anopheles stephensi*	Aqueous leaf filtrate from *Artemisia nilagirica*	1st−4th instar: 0.46; 0.35; 0.33; 0.21% Pupa: 0.16% LC_90:_ N/A	24 h	Nalini et al., 2017

*N/A- data not provided by the autor; LC_50_, Lethal Concentration that kills 50% of the exposed larvae; LC_90_, Lethal Concentration that kills 90% of the exposed larvae; AgNPs—T, prepared by heating; AgNPs—RT, prepared by non-heating*.

## Gaps, Obstacles, and Conclusions

The potential application of nano-based formulations in the field of arboviruses management was investigated by analyzing the number of publications in the last ten years. The results revealed that around 1000 articles were published worldwide, which an exponential trend in publication numbers after 2016, mainly for researches related to zika and chikungunya. Overall, many publications have shown that nanotechnology has led to rapid advancements in the development of pesticides, repellents, drug delivery system and diagnostic devices for arboviruses management. However, the clinical translational of nano-based formulations has some bottlenecks that hinders the broad acceptance and commercialization of these products (Hua et al., 2018; Soares et al., 2018). Also, it's already known, that nanomaterials, independently of their composition and method of production, have new properties, which are not observed by their bulk materials (Laux et al., 2018). These novel properties have brought innovative solutions due their flexibleness, responsiveness and possibility of functionalization, the last one, which is very useful for drug targeting delivery (Jeevanandam et al., 2018). However, the behavior, fate, bioavailability nanomaterials in the environment and toxicity of non-target organisms should be better understood the possible environmental impacts (Dinda, 2018).

Much debate still exists regarding the legislation of nanomaterials, which is in an early stage of development. In addition, there is a lack of a clear definition of nanomaterials, lack of standard methods for assessment of pharmacology, toxicology and efficacy evaluation of nano-based formulations and lack of worldwide network for gathering and sharing pertinent information. According to Schnell-Inderst et al. (2018) 12% of the documents related to the test of medical devices are written by academics and the regulators write the rest. In addition, there is a low number of experts in nanomaterials in regulatory agencies, resulting in the delayed development of these documents. Another issue is that FDA use the traditional regulatory frameworks to approve products, which contains nanomaterials (Jones et al., 2019). Nano-based formulations are evaluated by FDA using case-by-case approach, through combination product framework in order to determine regulatory framework will be used. Three main challenges should be addressed by FDA to boost the market of nano-based formulations: (i) development of a regulatory framework specific to nanomaterials; (ii) development of methods capable of characterize and quantify the toxicological impacts of nanomaterials, and (iii) deal with public acceptance and understanding through awareness programs and product labeling (Hua et al., 2018; Jones et al., 2019).

Also, economic issues can limiting the broad commercialization of nano-based formulations (Ventola, 2017; Jones et al., 2019). In comparison to conventional therapies, the production of a nano-based formulations required higher initial investments that will only be worth it from a business perspective if brings good opportunities for the pharmaceutical company, justifying their investment of R&D sector and reduce substantially health care costs (Benelli et al., 2017; Hua et al., 2018). Future research should explore the possibility of (i) determine the mechanism of action of nano-based formulations; (ii) understand the behavior and interaction of nanomaterial in complex biological matrix, such as human body; (iii) evaluate the possible toxicity and residual effects of nanomaterials in both target and non-target organisms; (iv) development of a international legislation; (v) create a standard definition of nanotechnology and nanomaterials, and (vi) standardized methods for establishing the risk-benefit of nanomaterials, in order to create large scale production of nanodevices to fight against of arboviruses as suggested in literature (Parisi et al., 2015; Kah et al., 2018a,b).

As conclusion, as indicated in this review, it is important to learn and appreciate the great potential offered by nanotechnology in relation to the development of new and more efficient tools and products. In this regard, it is extremely important that ongoing research involves all sectors: academia, industries, research centers, and government agencies, in order to turning this technology into a sustainable commercial reality and to create alternatives for detection and control of *Aedes aegypti*-borne diseases.

## Author Contributions

EC, JO, and LF proposed the structure of the manuscript. EC, JO, DA, CR, CB, VM, and RM wrote the manuscript. EC and LF revised the manuscript. All authors read and approved the final version of the manuscript.

### Conflict of Interest

The authors declare that the research was conducted in the absence of any commercial or financial relationships that could be construed as a potential conflict of interest.
